# Incidence of in-hospital cardiac arrest at general wards before and after implementation of an early warning score

**DOI:** 10.1186/s12873-021-00469-5

**Published:** 2021-07-07

**Authors:** Andreas Creutzburg, Dan Isbye, Lars S. Rasmussen

**Affiliations:** 1 Department of Anaesthesia, Centre of Head and Orthopaedics, Rigshospitalet – University of Copenhagen, Inge Lehmanns Vej 6, section 6011, DK-2100 Copenhagen, Denmark; 2 Faculty of Health and Medical Sciences, University of Copenhagen, Copenhagen, Denmark; 3 Department of Clinical Medicine, University of Copenhagen, Copenhagen, Denmark

**Keywords:** In-hospital cardiac arrest, Incidence, Early warning score, General wards

## Abstract

**Background:**

In order to reduce the incidence of in-hospital cardiac arrest (IHCA) at general wards, medical emergency teams (MET) were implemented in the Capital Region of Denmark in 2012 as the efferent part of a track and trigger system. The National Early Warning Score (NEWS) system became the afferent part. This study aims at investigating the incidence of IHCA at general wards before and after the implementation of the NEWS system.

**Material and methods:**

We included patients at least 18 years old with IHCA at general wards in our hospital in the periods of 2006 to 2011 (pre-EWS group) and 2013 to 2018 (post-EWS group). Data was obtained from a local database and the National In-Hospital Cardiac Arrest Registry (DANARREST). We calculated incidence rate ratios (IRR) for IHCA at general wards with 95% confidence interval (95% CI). Odds ratios (OR) for return of spontaneous circulation (ROSC) and 30-day survival were also calculated with 95% CI.

**Results:**

A total of 444 IHCA occurred before the implementation of NEWS at general wards while 494 IHCA happened afterwards. The incidence rate of IHCA at general wards was 1.13 IHCA per 1000 admissions in the pre-EWS group (2006–2011) and 1.11 IHCA per 1000 admissions in the post-EWS group (2013–2018). The IRR between the two groups was 0.98 (95% CI [0.86;1.11], *p* = 0.71). The implementation did not affect the chance of ROSC with a crude OR of 1.14 (95% CI [0.88;1.47], *p* = 0.32) nor did it change the 30-day survival with a crude OR 1.30 (95% CI [0.96;1.75], *p* = 0.09).

**Conclusion:**

Implementation of the EWS system at our hospital did not decrease the incidence rate of in-hospital cardiac arrest at general wards.

## Introduction

In-hospital cardiac arrest (IHCA) is a substantial burden for the patients, for their family and for the healthcare system in general [1]. However, throughout time, research in IHCA has received little attention compared to other high-risk in-hospital conditions even though IHCA is regarded a reversible condition [2] if caught in time. The critical role of early identification of patients with deviating vital signs is underlined in the European Resuscitation Council’s (ERC) guidelines. Here, the chain of survival states that an effective track and trigger system and staff education are needed to decrease the incidence of IHCA. Especially, patients admitted to unmonitored general wards are at risk of deteriorate unnoticed by the ward staff [3]. A deterioration of vital signs prior to an IHCA seems to occur in as many as 80% of patients [4]. Medical emergency team (MET) has been implemented to assist general wards as the efferent part of such a track and trigger system [5]. These teams help to intervene before patients become so ill that an IHCA happens or to initiate a discussion regarding end-of-life care planning and to deescalate treatment rather than to intensify it [6].

In 2012, the National Early Warning Score (NEWS) was developed by the Royal College of Physicians [7]. Hereby, standardising the more than 100 track and trigger systems used at the time [5]. The NEWS system is a systematic approach for general wards used for observation of patients. It is the afferent part of the track and trigger system [5]. The effectiveness of the system is dependent on systematic monitoring and critical, clinical assessment of patients. Furthermore, the whole response system only works if actions are taken upon deviating vital signs by the ward staff.

A Danish study by *Andersen* et al has estimated that 2500 IHCA happen at Danish hospitals annually. Furthermore, the 30-day survival after IHCA is estimated to be 27% [8]. The NEWS system was implemented in the Capital Region of Denmark on June 1st 2012 [9] followed by an escalation protocol (Table 1) in attempt to decrease deterioration of acutely ill patients and thereby decrease the risk of IHCA among patients admitted to general wards.

**Table 1:** National Early Warning Score (NEWS) used in the Capital Region of Denmark^,^ [10]. ^¶^: Alert (A), verbal (V), pain (P), unresponsive (U), ^»^MET: Medical Emergency Team

Vital sign	3	2	1	0	1	2	3
*Respiratory rate, min*^*− 1*^	< 9		9–11	12–20		21–24	> 24
*Arterial oxygen saturation, %*	< 92	92–93	94–95	> 95			
*Oxygen supplement* *L x min*^*− 1*^		Yes		No			
*Pulse rate, min*^*−1*^	< 41		41–50	51–90	91–110	111–130	> 130
*Systolic blood pressure* *mmHg*	< 91	91–100	101–110	111–219			> 219
*Mental status, AVPU*^*¶*^				A			V, P, U
*Temperature, °C*	< 35.1		35.1–36.0	36.1–38.0	38.1–39.0	> 39.0	
**EWS algorithm**							
*NEWS*	*Frequency of monitoring*	*Clinical response according to escalation protocol*					
0–1	Minimum 12 hourly ± 1 h	Continue EWS monitoring minimum 12 hourly Frequency can be increased					
2	Minimum 6 hourly ± 30 min	Nursing staff ABCDE optimise					
3–5	Minimum 4 hourly	Nursing staff ABCDE optimise AND informs the on-call physician The on-call physician makes a documented treatment plan					
6	Minimum 4 hourly	Nursing staff ABCDE optimise AND contacts the on-call physician immediately The on-call physician assesses AND makes a documented treatment plan					
7–8	Minimum 1 hourly	Nursing staff ABCDE optimise AND contacts the on-call physician immediately – attends within 30 min The on-call physician assesses AND makes a documented treatment plan immediately Consider contacting the MET^»^ or anaesthesiologic assistance					
> 9	Minimum 0.5 hourly	Nursing staff ABCDE optimise AND contacts the on-call physician immediately – attends within 15 min The on-call physician assesses AND makes a documented treatment plan immediately Consider contacting the MET^»^ or anaesthesiologic assistance					

This study aims to assess the incidence of IHCA at general wards before and after the implementation of the NEWS system in 2012.

## Methods

This was conducted as a retrospective quality assurance study based on data from our institution. Data about the IHCA events and return of spontaneous circulation (ROSC) was obtained from The Danish In-Hospital Cardiac Arrest Registry (DANARREST) from 2017 and forward, while earlier data was obtained from a local database. From DANARREST, data on personal identification number (CPR number), sex, ROSC and 30-day survival were provided [11]. The IHCA were divided into two groups relative to the implementation of the NEWS system, which was implemented on 1st of June 2012 (Pre-EWS (2006–2011) versus Post-EWS (2013–2018)) [9]. All patients suffering an IHCA in 2012 were excluded to avoid bias.

### Study population

Data was collected in the period of 1st of January 2006 to 31st of December 2018 apart from 2012. We included all cardiac arrest team activations in this period. We excluded patients under the age of 18 years and patients admitted to wards not using the NEWS system such as intensive care units, operating theatres, cardiac catherization labs, etc. In the data analysis, we only included patients who had cardiac arrest at general wards according to the definition by the ERC [3] with indication for resuscitation.

### Setting

The study was undertaken at Copenhagen University Hospital, Rigshospitalet, a tertiary 1200-bed hospital in central Copenhagen, Denmark. Our institution offers highly specialised treatment for the Eastern part of Denmark, including cardiac surgery, coronary angiography, neurosurgery, and organ transplantation. The NEWS consists of measurements of respiratory rate, arterial oxygen saturation by pulse oximetry, pulse rate, systolic blood pressure, mental status, and temperature. If a parameter deviates, it triggers one or more points. The NEWS score accounts for the sum of all points. At our hospital, the NEWS system has an algorithm for the clinical response by the ward staff and when to active the trigger part of the system according to the measured NEWS (Table 1). During the study period, there was no major changes in the set-up of in-hospital cardiac arrest treatment at our institution which has followed the applicable guidelines of European Resuscitation Council since 2000.

### Statistical analysis

Descriptive statistics were used to describe characteristics of patients suffering IHCA. *P*-values < 0.05 were considered significant. We aimed to detect a 20% relative risk reduction in the incidence of IHCA comparing the pre-EWS (2006–2011) group with post-EWS group (2013–2018).

For the primary analysis, the incidence rates of the pre-EWS group and the post-EWS group were compared by the incidence rate ratio (IRR) with 95% confidence intervals. The incidence rates were reported as IHCA per 1000 admissions at general wards only at our institution which was based on data from a local database. The number of admissions varied during the study period and the incidence rates were calculated on a year-to-year basis to avoid bias.

The secondary outcomes, ROSC and 30-day survival, were expressed as Odds ratios (OR) with 95% confidence intervals. These were adjusted for age, sex, and primary rhythm categorised as shockable or non-shockable by Mantel-Haenszel method.

All analyses were performed using R version 3.6.2 (R Project for Statistical Computing) and RStudio version 1.2.5033.

## Results

**Fig. 1:**
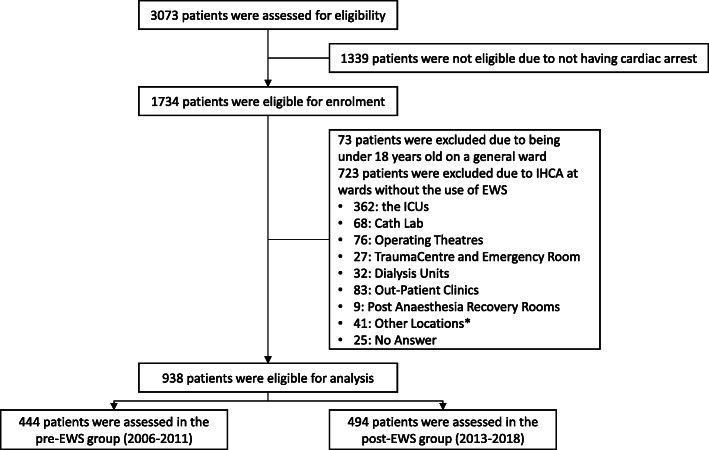
Study profile of in-hospital cardiac arrest from 2006 to 2018, excluding 2012. ICU: Intensive Care Unit. Cath lab: Cardiac Catherization Laboratory. *: Radiology, Offices, Psychiatric department, not admitted to a specific ward

During the two time periods, the cardiac arrest team was activated 3073 times overall. In total 1734 confirmed IHCA with the need for resuscitation were identified. Of these, we excluded 723 IHCA due to location, and 73 IHCA due to age. The remaining 938 IHCA in general wards were analysed. Of these, 444 IHCA occurred before the 1st of June 2012 (pre-EWS group (2006–2011)) while 494 IHCA happened after that date (post-EWS group (2013–2018)) (Fig. 1).

### Patient’s characteristics

The mean age was 66 years (± 12.2 years) versus 67 years (± 13.1 years) (*p* = 0.73). The distribution of sex was similar in the two groups (64.6% versus 69.2%). The primary rhythm was most commonly Pulseless Electrical Activity (PEA) in both groups but differed between the two time periods (35.4% versus 44.1%, *p* = 0.03) (Table 2).

**Table 2:** Characteristics of in-hospital cardiac arrest at general wards before and after implementation of National Early Warning Score (NEWS) at Rigshospitalet in the two periods of 2006 to 2011 and 2013 to 2018

Characteristics	Pre-EWS (2006–2011) *N* = 444 (%)	Post-EWS (2013–2018) *N* = 494 (%)	*P*-value
*Demographics*			
Age (year)	66 (± 12.2)	67 (± 13.1)	0.73
Male gender	287 (64.6)	342 (69.2)	0.15
*Primary rhythm*			
VT/VF - Shockable rhythm	100 (22.5)	89 (18.0)	0.03
Pulseless Electrical Activity	157 (35.4)	218 (44.1)	
Asystole	145 (32.7)	154 (31.2)	
Unknown	42 (9.5)	33 (6.7)	

### Primary outcome

The incidence rate of IHCA at general wards was 1.13 IHCA per 1000 admissions in the pre-EWS group (2006–2011). In the post-EWS group (2013–2018), the incidence rate was 1.11 IHCA per 1000 admissions. The IRR between the two groups was 0.98 (95% CI [0.86;1.11], *p* = 0.71) (Table 3).

**Table 3:** The incidence rate ratio (IRR) of in-hospital cardiac arrest and the crude and the Mantel-Haenszel adjusted Odds ratios (OR) of return of spontaneous circulation (ROSC) and 30-day survival (30 DS) for the confounders age, sex and primary rhythm categorised as shockable or non-shockable. °: 7 patients in the post-EWS groups did not obtain ROSC due to being treated with ECMO in connection to the resuscitation

	Result		P-value
Incidence rate ratio	0.98	[0.86;1.11]	0.71
OR_ROSC_°	1.14	[0.88;1.47]	0.32
Adjusted OR_ROSC_ °	1.24	[0.92;1.68]	0.10
OR_30 DS_	1.30	[0.96;1.75]	0.09
Adjusted OR_30 DS_	1.64	[1.15;2.35]	0.004

### Secondary outcome

**Fig. 2:**
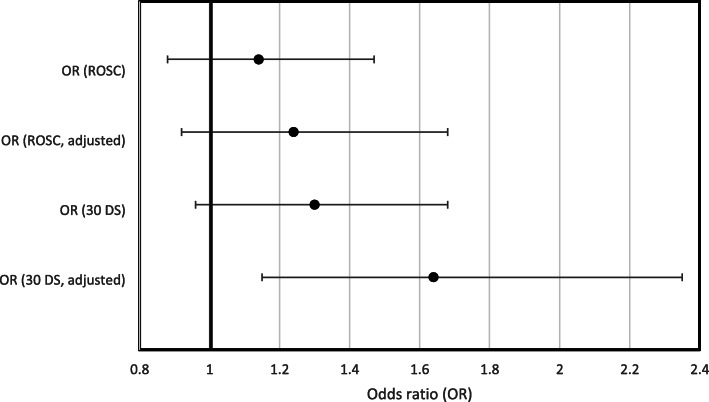
Forest plot of ORs for the secondary outcome. Both crude and adjusted ORs of return of spontaneous circulation (ROSC) and 30-days survival (30 DS) are shown

When comparing the pre-EWS group (2006–2011) with the post-EWS group (2013–2018), OR of ROSC was 1.14 (95% CI [0.88;1.47], *p* = 0.32). Adjusting for age, sex, and primary rhythm gave an OR of 1.24 (95% CI [0.92;1.68], *p* = 0.10) (Table 3, Fig. 2).

The OR of 30-day survival was 1.30 (95% CI [0.96;1.75], *p* = 0.09). When adjusting the 30-day survival OR for age, sex, and primary rhythm, the OR was 1.64 (95% CI [1.15;2.35], *p* = 0.004) (Table 3, Fig. 2).

## Discussion

In our study, we found that the rate of IHCA among adult patients in general wards in our hospital did not decrease after implementation of an EWS system. In contrast, the point estimate for the incidence rate ratio was 0.98 (95% CI [0.86;1.11], *p* = 0.71) for the post-EWS (2013–2018) versus the pre-EWS (2006–2011) time period.

OR of ROSC was 1.14 (95% CI [0.88;1.47], *p* = 0.32). For 30-day survival, the OR was 1.30 (95% CI [0.96;1.75], *p* = 0.09). The *p*-values of adjusted ORs for age, sex, and primary rhythm indicate that ROSC was not affected, while 30-day survival significantly improved.

*Roberts* et al showed that a medium or high EWS was correlated to an increased risk IHCA [1]. However, another study has shown that the implementation did not affected the incidence or even an increase in the incidence rate occurred [12], as we found in our study. The continuous monitoring of patients with the NEWS system is a staff-intensive assignment and it was expected that a beneficial effect could be detected after implementation. Even though, it has not been possible to show a positive effect on IHCA incidence at our institution, the NEWS system has given nursing staff and physicians clinical tools for handling the acutely deteriorating patients with the escalation protocol [1]. The incidence of IHCA at our institution is somewhat lower than reported in other studies in western countries where incidence of IHCA ranges between 1.5 to 10.2 per 1000 admissions [13, 14]. One possible explanation for the low incidence in our study could be due to the exclusion of cardiac arrest in intensive care units, trauma units, and cardiac catherization labs where a fair number of IHCA happen (Fig. 1).

Even though, NEWS can be used to stratify patients at risk of IHCA, it does not consider the increasing age in the population (Table 1). Physiological parameters are known to change in the elderly population [15]. Age has been identified as an independent risk factor for development of cardiac arrest [16]. Studies have shown that the accuracy of EWS systems decrease with increasing age, especially when age is above 65 years. This might have contributed to the non-significant result of our study.

Several other problems have been identified as reasons for the constant incidence rate. *Petersen* et al found that under-staffing and time constrains were reasons for not adhering to the protocol [17]. Provided that the NEWS protocol was followed, and vital signs were monitored continuously, the system only works if actions are taken upon deviating measurements. Nurses should contact the on-call physician according to the escalation protocol if the NEWS score was 3 in a single variable (Table 1). That did not always happen and that seemed to be related to lack of experience with acutely ill patients or bad experiences with the MET previously [18]. Hereby, treatment of deteriorating patients was delayed, and the risk of cardiac arrest was increased. Problems with assistance from the MET have also been identified [17] even though, the latest guidelines of ERC specifically underline the importance of teamwork between staff at general wards and the MET [3]. These factors could, perhaps, explain why a decrease in the incidence of IHCA was not seen after the implementation of the NEWS. In addition, the number of admissions has increased during the post-EWS (2013–2018) time period and this may also affect the primary result of the study, drawing the conclusion towards the null.

An increase in OR of ROSC and 30-days survival was seen. With the NEWS system ward staff are giving a systematic approach to the acutely deteriorating patient. This could mean that if the monitoring frequency was high enough some patients could be caught right after the cardiac arrest and thereby improving their chance of survival. However, this is speculative, and the system is not intended to improve outcome after IHCA. ROSC and 30-day survival are more likely to be influenced by changing guidelines, response time and training level of the cardiac arrest team, the ward staff, and post resuscitation care. Our institution has followed these guidelines since 2000, and all members of the cardiac arrest team are regularly trained and recertified regarding resuscitation according to the newest guidelines.

### Strengths and limitations

This study has compared periods of 6 years before and after the implementation of the NEWS system at our institution. This fairly long time period should provide a robust assessment of the effect. Furthermore, we excluded the year of implementation. Missing data was not a major source of error, as data was obtained from DANARREST. In 2017, only 4% of the registered IHCA had missing data [11].

The study was conducted as a retrospective study where data from a period before and after the implementation is compared. Therefore, organisational changes have not been included in the analysis which include increased focus on patient safety regarding earlier treatment of sepsis, changes in medication, etc. Furthermore, it was undertaken as a single centre study at a tertiary hospital, which limits the generalisability of the results to other institutions with another patient population.

One of the limitations of this study is that it was not possible to describe the risk of in-hospital cardiac arrest prior in the patient population since data regarding DNAR-orders are not available. Nor was it possible to extract data on overall in-hospital mortality. However, there were no major changes in hospital size, number of specialties, catchment area, or population during the study period.

## Conclusion

This study suggests that the implementation of the EWS system at our hospital did not decrease the incidence rate of in-hospital cardiac arrest at general wards.

## Data Availability

The datasets used and analysed during the current study are available from the corresponding author on reasonable request.
